# Safety without the counter: transparency of online safety information for women’s over-the-counter medicines across markets

**DOI:** 10.1080/20523211.2026.2707406

**Published:** 2026-08-28

**Authors:** Samar J. Melhem, Hamzeh Al Momani, Rimal Mousa, Eman Massad, Yazan Al Rashdan, Sameh Alzubiedi, Violet Kasabri, Rula Darwish, Nailya Bulatova

**Affiliations:** a Department of Biopharmaceutics and Clinical Pharmacy, School of Pharmacy, University of Jordan, Amman, Jordan; b Department of Pharmaceutics and Pharmaceutical Technology, School of Pharmacy, University of Jordan, Amman, Jordan; c Department of Health Economics and Health Management, Public Health Institute, University of Jordan, Amman, Jordan

**Keywords:** Women’s health, over-the-counter medicines, e-pharmacy, medication safety, health information transparency, digital pharmacy, self-care

## Abstract

**Background:**

The rapid expansion of e-pharmacy platforms has shifted over-the-counter (OTC) medicine selection from physical pharmacies to digital environments, where consumers increasingly rely on online product pages for safety-critical information. For women’s health OTC products, this shift raises particular concerns due to pregnancy- and lactation-related risks, frequent self-care use, and reduced access to professional counseling at the point of purchase. Despite growing reliance on e-pharmacies, evidence on the completeness of online safety information across regulatory contexts remains limited.

**Methods:**

A cross-sectional observational study assessed the transparency of safety information for women’s health OTC medicines and supplements sold on licensed e-pharmacy platforms across seven markets (United States, United Kingdom, Canada, Australia; United Arab Emirates, Saudi Arabia, Qatar). Product pages were evaluated using a standardized 12-item transparency framework capturing essential safety elements. Comparative analyses employed nonparametric tests and multivariable regression to identify regional, platform-level, and product-related predictors of transparency.

**Results:**

A total of 119 products were analyzed. The mean Transparency Score was 6.25 (SD 1.81) out of 12, indicating moderate completeness. Critical elements, including expiry date, batch or lot number, and drug interaction information, were rarely disclosed. While overall transparency differed modestly by region, warnings and precautions were significantly less likely to appear on Middle Eastern platforms. Independent or regional pharmacies demonstrated lower transparency than chain platforms. Pregnancy and lactation guidance was inconsistently provided outside pregnancy-specific categories.

**Conclusion:**

Licensed e-pharmacy platforms frequently provide incomplete safety information for women’s health OTC products, with meaningful variation by market region, platform type, and availability of supplementary digital resources. Transparency deficits are embedded within regulated digital pharmacy systems rather than limited to illicit sellers. These findings underscore the need for harmonized regulatory standards, mandatory digital safety disclosures, and strengthened platform accountability to support safe, informed self-care in online pharmacy environments.

## 1. Background

The consumerization of digital health has reshaped how medicines and health products are accessed, evaluated, and used, with e-pharmacy platforms increasingly functioning not only as retail outlets but also as primary sources of medication-related information. This shift accelerated during the COVID-19 pandemic, normalizing remote procurement and intensifying demand for home delivery, convenience, and cross-platform price comparison (Fittler et al., 2024; Wickware, 2021). In England, dispensing volumes from distance-selling pharmacies rose sharply between 2019 and 2020, reflecting the migration of safety-critical medication decisions to online environments where professional counseling is not routinely available (Wickware, 2021). Similar trends have been reported internationally, alongside persistent limitations in consumers’ ability to identify legitimate online pharmacies or critically appraise safety information, increasing exposure to misleading content and unsafe sellers (Fittler et al., 2024; Gharaibeh et al., 2023; Limbu & Huhmann, 2024).

Within e-pharmacy settings, medication safety is closely tied to information transparency. For over-the-counter (OTC) products, consumers are expected to recognize symptoms appropriate for self-care, select suitable therapies, and adhere to dosing instructions and warnings without mandatory professional input. These tasks depend on the clear presentation of essential safety information, including active ingredients, indications, dosing, contraindications, warnings, adverse effects, interactions, and guidance for special populations. Regulatory authorities have long emphasized standardized consumer-facing information for nonprescription medicines, exemplified by the U.S. Food and Drug Administration’s Drug Facts labeling framework (U.S. Food and Drug Administration, 2009, 2024). As purchasing increasingly occurs within digital storefronts, however, a disconnect has emerged between information required on physical packaging and what is encountered during online selection. Consumers may complete transactions without accessing full labels, patient information leaflets, or prominent warnings, and although initiatives promoting electronic product information underscore the importance of authorized digital labeling, implementation remains uneven across markets and product categories (European Medicines Agency, 2023).

The cross-border structure of online medicine sales further complicates transparency. Illicit pharmacies and deceptive redirection practices persist globally, facilitated by search-engine dynamics and the ease with which illegitimate platforms can replicate regulated services (Fittler et al., 2022). Regulatory responses have largely focused on signaling legitimacy, including the European Union’s common logo system under Directive 2011/62/EU (European Commission, 2014), professional registration and distance-selling standards in Great Britain (General Pharmaceutical Council, 2025), and digital pharmacy accreditation programs in the United States (National Association of Boards of Pharmacy, 2024). While these mechanisms support governance and consumer trust, they do not ensure that safety-critical information is consistently available at the product-page level, particularly for OTC medicines and supplements, where oversight has historically prioritized authorization rather than point-of-purchase information completeness.

Women’s health OTC products represent a domain in which deficiencies in online safety information carry heightened consequences. Across the life course, women face distinct medication-safety considerations related to reproductive health, pregnancy and lactation, perimenopause and menopause, and greater exposure to polypharmacy. Pharmacovigilance and epidemiologic evidence consistently report higher rates of adverse drug reactions among women across many drug classes (Hendriksen et al., 2021; Sportiello & Capuano, 2024). In online purchasing environments with limited access to real-time pharmacist consultation, the absence of explicit warnings, contraindications, and interaction information may therefore disproportionately affect women. OTC products are commonly used for menstrual symptoms, genitourinary conditions, iron deficiency, and general wellness, often alongside hormonal contraception or other therapies, creating safety considerations that may not be apparent without clear guidance. Evaluations of emergency contraception information further illustrate how variability in online content quality and readability can influence time-sensitive decision-making (Agrawal et al., 2021).

Pregnancy and lactation further amplify the risks associated with incomplete safety communication. OTC medicine use during pregnancy is widespread, and evidence indicates that many pregnant individuals use nonprescription products with limited understanding of pregnancy-specific risks, frequently influenced by the assumption that OTC status implies safety (Thiruchelvam et al., 2025). Studies from Gulf region settings similarly document both OTC and prescription medicine use during pregnancy and highlight reliance on information sources that are not consistently clinically grounded (Alyami et al., 2023). In this context, explicit pregnancy and breastfeeding guidance, or clear advice to seek professional consultation, is central to safe use rather than supplementary.

Despite the expansion of e-pharmacy research, important gaps persist. Much of the literature has focused on illegitimate sellers, consumer trust, or system-level compliance, offering limited insight into whether consumers encounter the core safety information required for informed OTC self-care at the moment of online selection. Evidence from studies of written medicine information continues to demonstrate challenges in comprehension and usability (Raynor et al., 2007), and compliance with local labeling requirements does not necessarily ensure interpretability or alignment with international expectations (Online pharmacies: A double-edged sword, 2025). In digital retail environments, where marketing priorities often favor brevity, incomplete or inconsistently displayed safety information may further undermine safe use. Cross-market comparative evidence remains scarce, particularly for women’s health OTC products, which have rarely been examined as a distinct category despite their widespread use and relevance across regulatory contexts.

Accordingly, this cross-sectional observational study evaluates the transparency of essential safety information for women’s health OTC medicines and supplements at the point of online purchase across seven markets, encompassing Middle Eastern (United Arab Emirates, Saudi Arabia, Qatar) and Western (United States, United Kingdom, Australia, Canada) contexts. Transparency is defined as the presence or absence of prespecified safety information elements on product pages and linked resources. The study aims to: (1) quantify the availability of essential safety information; (2) compare transparency across regulatory contexts; (3) identify systematically missing elements by product category and market; and (4) examine platform-level characteristics associated with variation in transparency. By generating cross-market benchmarks using a standardized assessment framework, this study provides an empirical basis for targeted regulatory and practice interventions to strengthen medication safety in digital pharmacy environments.

## 2. Methods

### 2.1. Study design and setting

A cross-sectional observational study evaluated the transparency of safety information for women’s health over-the-counter (OTC) medicines and supplements on licensed e-pharmacy platforms. Data were collected over an 11-week period (October–December 2025) and limited to publicly accessible information available at the point of online purchase. The study involved no human participants, patient-level data, or interaction with consumers or platform operators.

Seven national markets were selected for comparative analysis across regulatory contexts. Middle Eastern markets included the United Arab Emirates, Saudi Arabia, and Qatar; Western markets comprised the United States, the United Kingdom, Australia, and Canada.

### 2.2. Search strategy and identification of E-pharmacy platforms

Licensed e-pharmacy platforms were identified through systematic, market-specific internet searches conducted by the principal investigator. Standardized search terms reflecting typical consumer behavior were applied consistently across markets. English-language terms included ‘online pharmacy [country],’ ‘buy OTC products online [country],’ and ‘e-pharmacy [country],’ while equivalent Arabic-language terms (e.g. ‘صيدلية إلكترونية,’ ‘شراء أدوية أونلاين’) were used for Middle Eastern markets.

For each market, the first 30 search results returned by the search engine were screened. Platforms were logged and assessed against predefined eligibility criteria. Eligible platforms were licensed or registered online pharmacies or healthcare retailers that offered direct-to-consumer e-commerce functionality, provided English or Arabic interfaces, sold women’s health OTC products, and were operationally accessible at the time of data collection. Platforms were excluded if they were unlicensed or rogue sellers, prescription-only or information-only websites, lacked an English or Arabic interface, or were inaccessible due to geographic or technical restrictions.

Overall, 52 platforms were identified, of which 37 met all eligibility criteria and were included in the sampling frame. The complete search strategy is provided in Supplementary File 1.

### 2.3. Product selection and eligibility

Within each eligible platform, women’s health OTC products were systematically identified using platform category navigation and internal search functions. Standardized product search terms were applied across five predefined categories: menstrual health, menopausal health, pregnancy and prenatal care, reproductive health, and women’s supplements.

Products were eligible if they were nonprescription medicines or supplements marketed specifically for women’s health and intended for oral or topical use. Exclusion criteria included prescription-only status (n = 3), classification as medical devices (n = 4), lack of women’s health–specific marketing (n = 5), out-of-stock status at data collection (n = 4), or duplication within the same platform (n = 3).

**Figure 1.**
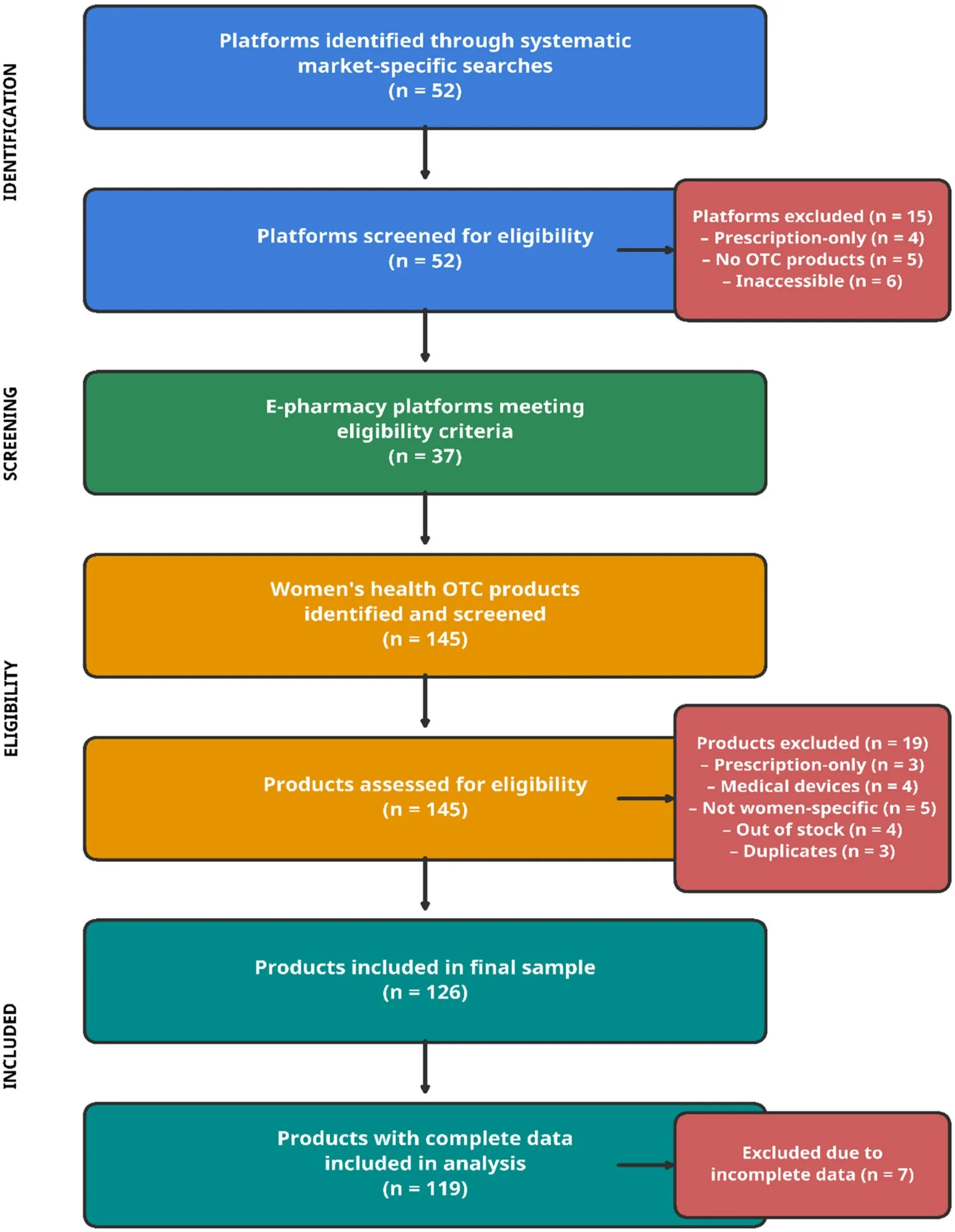
Flow diagram of e-pharmacy platform and product selection process.

A total of 145 products were screened. Nineteen were excluded based on eligibility criteria, yielding 126 products. Following data extraction, seven additional products were excluded because of incomplete safety information, resulting in a final analytic sample of 119 products. The platform and product selection process is summarized in Figure 1.

### 2.4. Data extraction instrument and variables

A structured data extraction instrument was developed based on international regulatory standards for OTC medicine labeling (European Commission, 2009; U.S. Food and Drug Administration, 2020), established frameworks for consumer medication information (Koo et al., 2003; Raynor et al., 2007), and expert input from the research team. The instrument assessed the presence or absence of twelve safety information elements essential for informed consumer decision-making: active ingredients; indications or uses; dosing instructions; contraindications; side effects; warnings or precautions; drug interactions; pregnancy and lactation guidance; storage instructions; expiry date; manufacturer information; and batch or lot number.

Each element was coded dichotomously (1 = present; 0 = absent). The primary outcome, the Transparency Score, was calculated as the sum of present elements (range 0–12) and categorized for descriptive interpretation as low (0–4), moderate (5–8), or high (9–12). A secondary binary outcome, Complete Pregnancy/Lactation Guidance, was defined as the presence of explicit guidance for both pregnancy and lactation versus incomplete or absent guidance.

Covariates included market region, women’s health product category, platform type (chain pharmacy, independent or regional pharmacy, online-only pharmacy), availability of a downloadable patient information leaflet, and presence of an interactive decision-support tool. Product identifiers (name, brand, URL) and date of data collection were recorded to support auditability.

### 2.5. Data collection procedures and quality assurance

Data collection followed a standardized protocol to ensure consistency and reproducibility. For each product, the principal investigator reviewed the main product page and all linked or downloadable resources to extract safety information, documenting the location of each element (e.g. main page, expandable section, downloadable leaflet).

Screenshots were retained for verification. Approximately 10% of products were independently double-coded by a second reviewer to assess coding consistency, with discrepancies resolved by consensus. All data were entered into a structured Microsoft Excel database developed for the study.

### 2.6. Sample size calculation

The minimum required sample size was determined a priori using the standard formula for comparing two independent means (Chow et al., 2017). Assuming a pooled standard deviation of 2.5, a minimum meaningful difference of 1.5 points in the Transparency Score, 80% power, and a two-sided α of.05, approximately 44–45 products per group were required (Rosner, 2015). This corresponded to a medium standardized effect size (Cohen’s d = 0.60; Cohen, 1988).

To account for potential exclusions or incomplete records, a 15% inflation factor was applied, yielding a target sample size of approximately 52 products per group (Lachin, 1981). Sampling continued until this target was met or exceeded in both regions.

### 2.7. Statistical analysis

Statistical analyses were conducted using IBM SPSS Statistics for Windows (Version 29.0). Statistical significance was defined a priori as a two-tailed *p* value < .05.

Descriptive statistics summarized product-, platform-, and market-level characteristics. Continuous variables were reported as means with standard deviations or medians with interquartile ranges, and categorical variables as frequencies and percentages.

Normality of the Transparency Score distribution was assessed using histograms, Q–Q plots, and the Shapiro–Wilk test and was violated; nonparametric methods were therefore applied. Regional differences were assessed using the Mann–Whitney U test with rank-biserial correlation as the effect size. Differences across product categories were evaluated using the Kruskal–Wallis H test, with Dunn’s post hoc tests and Bonferroni adjustment when appropriate, and effect size quantified using epsilon-squared (ϵ²).

Associations between market region and individual safety information elements were examined using χ² tests or Fisher’s exact tests when expected cell counts were <5, with odds ratios and 95% confidence intervals reported. *P* values were adjusted for multiple testing using Bonferroni correction and the Benjamini–Hochberg procedure.

Predictors of overall transparency were evaluated using multiple linear regression, with Transparency Score as the dependent variable and market region, product category, and platform type as covariates. Model assumptions were assessed, including multicollinearity using variance inflation factors. Binary logistic regression was conducted in parallel to identify predictors of complete pregnancy and breastfeeding guidance.

### 2.8. Ethical considerations

The study analyzed publicly accessible information from e-pharmacy websites and involved no human participants, personal data, or patient-level information; formal ethical approval was therefore not required. Data were collected without interaction with platform operators or consumers, and findings are reported in aggregate without identifying individual platforms.

## 3. Results

### 3.1. Sample characteristics

The final analytic sample comprised 119 unique women’s health over-the-counter (OTC) products identified on licensed e-pharmacy platforms across seven markets. Western markets contributed 77 products (64.7%) (United States, United Kingdom, Canada, Australia), while Middle Eastern markets contributed 42 products (35.3%) (United Arab Emirates, Saudi Arabia, Qatar).

Products were distributed across five predefined women’s health categories, with menstrual health products representing the largest proportion, followed by women’s supplements and menopausal health products. Most products were sold through chain pharmacy platforms, with smaller proportions offered by independent/regional pharmacies and online-only platforms.

### 3.2. Overall transparency and availability of safety information elements

Across the full sample, the mean Transparency Score was 6.25 (SD 1.81) out of a maximum of 12, with a median score of 6, indicating moderate overall completeness of safety information at the point of online purchase. Most products fell within the moderate transparency range (73.1%), while 18.5% were classified as low transparency and 8.4% as high transparency (Table 1), demonstrating substantial heterogeneity in information provision.

**Table 1.** Sample characteristics by market region (N = 119).

Characteristic	Overall (N = 119)	Middle Eastern (n = 42)	Western (n = 77)
Transparency Score, mean (SD)	6.25 (1.81)	5.71 (2.02)	6.55 (1.63)
Transparency level, n (%)			
Low (0–4)	22 (18.5)	12 (28.6)	10 (13.0)
Moderate (5–8)	87 (73.1)	28 (66.7)	59 (76.6)
High (9–12)	10 (8.4)	2 (4.8)	8 (10.4)
Product category, n (%)			
Menstrual health	29 (24.4)	7 (16.7)	22 (28.6)
Women’s supplements	26 (21.8)	9 (21.4)	17 (22.1)
Menopausal health	26 (21.8)	6 (14.3)	20 (26.0)
Pregnancy & prenatal	22 (18.5)	10 (23.8)	12 (15.6)
Reproductive health	14 (11.8)	8 (19.0)	6 (7.8)
Other	2 (1.7)	2 (4.8)	0(0.0)
Platform type, n (%)			
Chain pharmacy	82 (68.9)	34 (81.0)	48 (62.3)
Independent/regional	23 (19.3)	8 (19.0)	15 (19.5)
Online-only pharmacy	14 (11.8)	0 (0.0)	14 (18.2)

Marked variability was observed across individual safety information elements. Indications/uses (99.2%) and active ingredient information (97.5%) were nearly universally available. Dosing instructions were also frequently provided, though less consistently. In contrast, several safety-critical and traceability elements were rarely disclosed, including expiry date (6.7%), batch or lot number (7.6%), and drug interaction information (10.9%).

**Figure 2.**
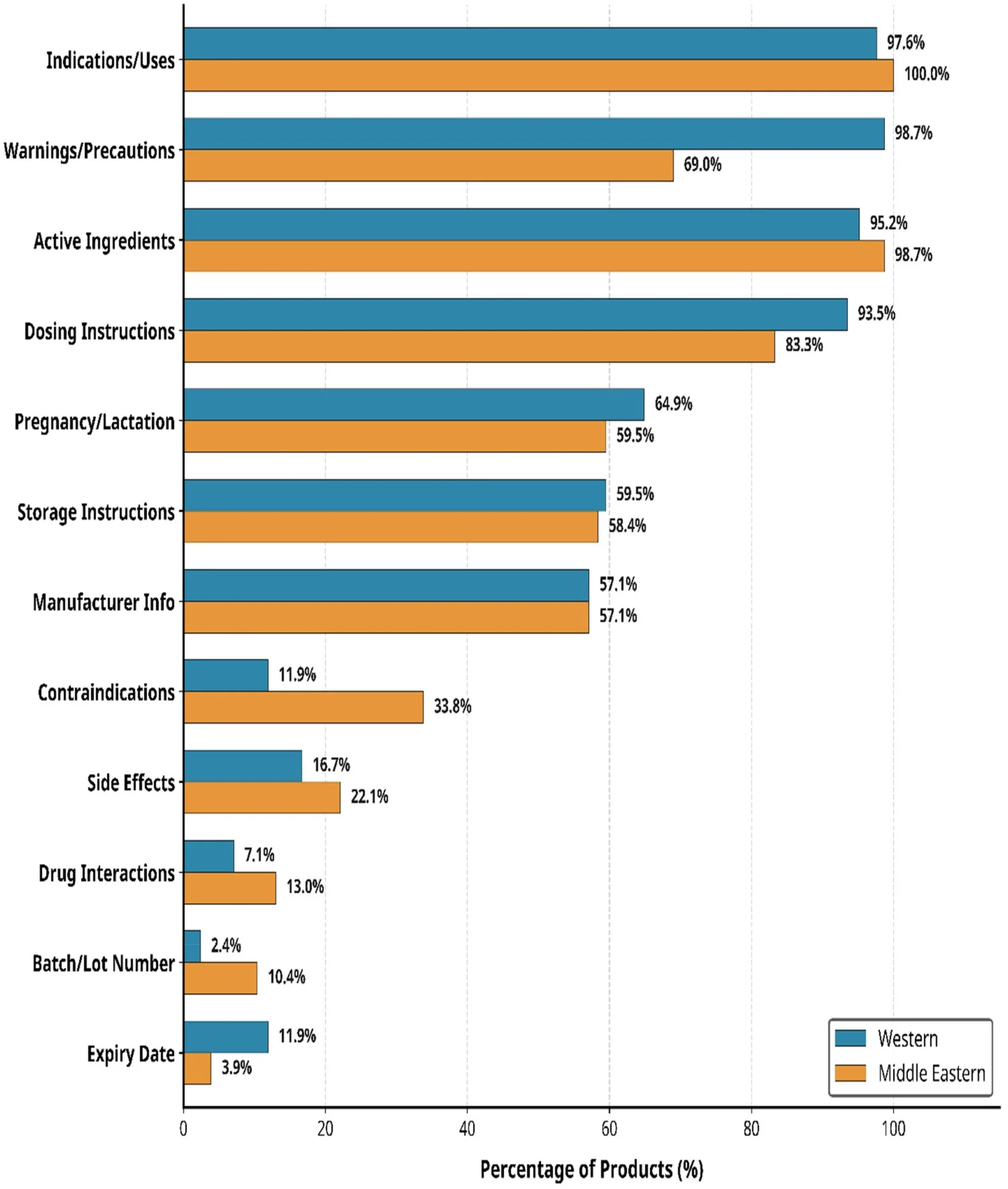
Availability of safety information elements by market region.

When stratified by market region, disclosure patterns differed by element rather than overall presence (Figure 2). High-level product descriptors were commonly available in both regions, whereas warnings and precautions were less consistently displayed on products sold through Middle Eastern platforms. Low availability of drug interaction information, expiry dates, and batch/lot numbers was observed across both regions, suggesting system-level rather than market-specific omissions.

**Figure 3.**
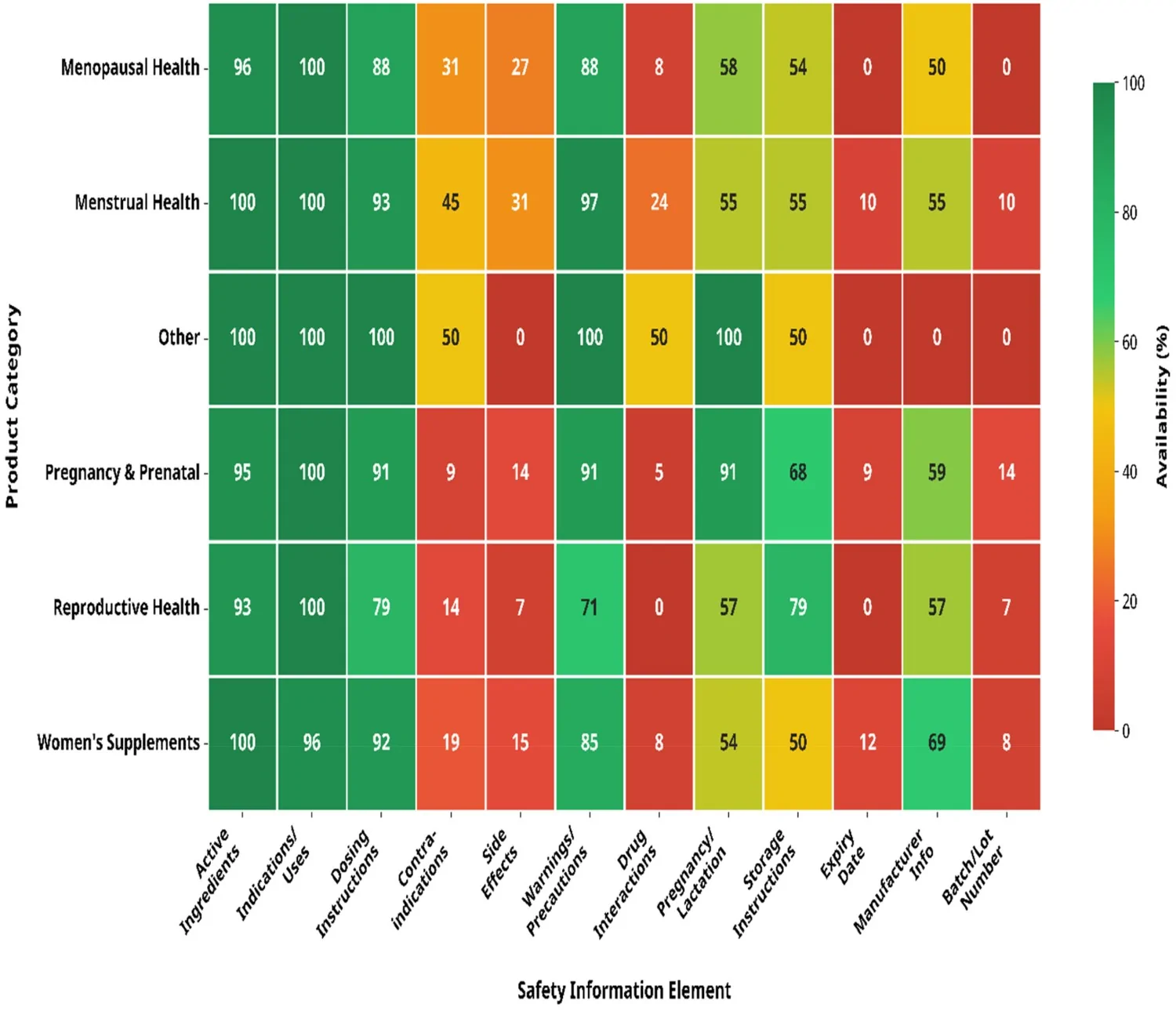
Heatmap of safety information availability across women’s health OTC product categories.

Element availability also varied across product categories (Figure 3). Pregnancy and prenatal products most consistently included pregnancy and lactation guidance, whereas such guidance was inconsistently provided for all other categories. Across categories, traceability elements and drug interaction information remained uniformly sparse.

### 3.3. Regional differences in transparency scores

Products marketed through Western e-pharmacy platforms demonstrated higher overall transparency than those sold through Middle Eastern platforms (M = 6.55 vs. 5.71). Given the non-normal distribution of scores, the Mann–Whitney U test indicated a trend toward higher transparency in Western markets (U = 1290.0, *p* = 0.062), with a small effect size (rank-biserial r = 0.20).

Western products were more tightly clustered around moderate scores, whereas Middle Eastern products showed greater dispersion and a longer lower tail.

### 3.4. Element-Specific regional disparities

Although overall Transparency Scores did not differ significantly by region, element-level analyses revealed a selective and clinically relevant disparity. After correction for multiple comparisons, warnings and precautions were significantly less likely to be displayed on Middle Eastern platforms (69.0%) compared with Western platforms (98.7%), corresponding to substantially lower odds of disclosure (OR = 0.04, 95% CI 0.01–0.32; *p* < 0.001).

**Figure 4.**
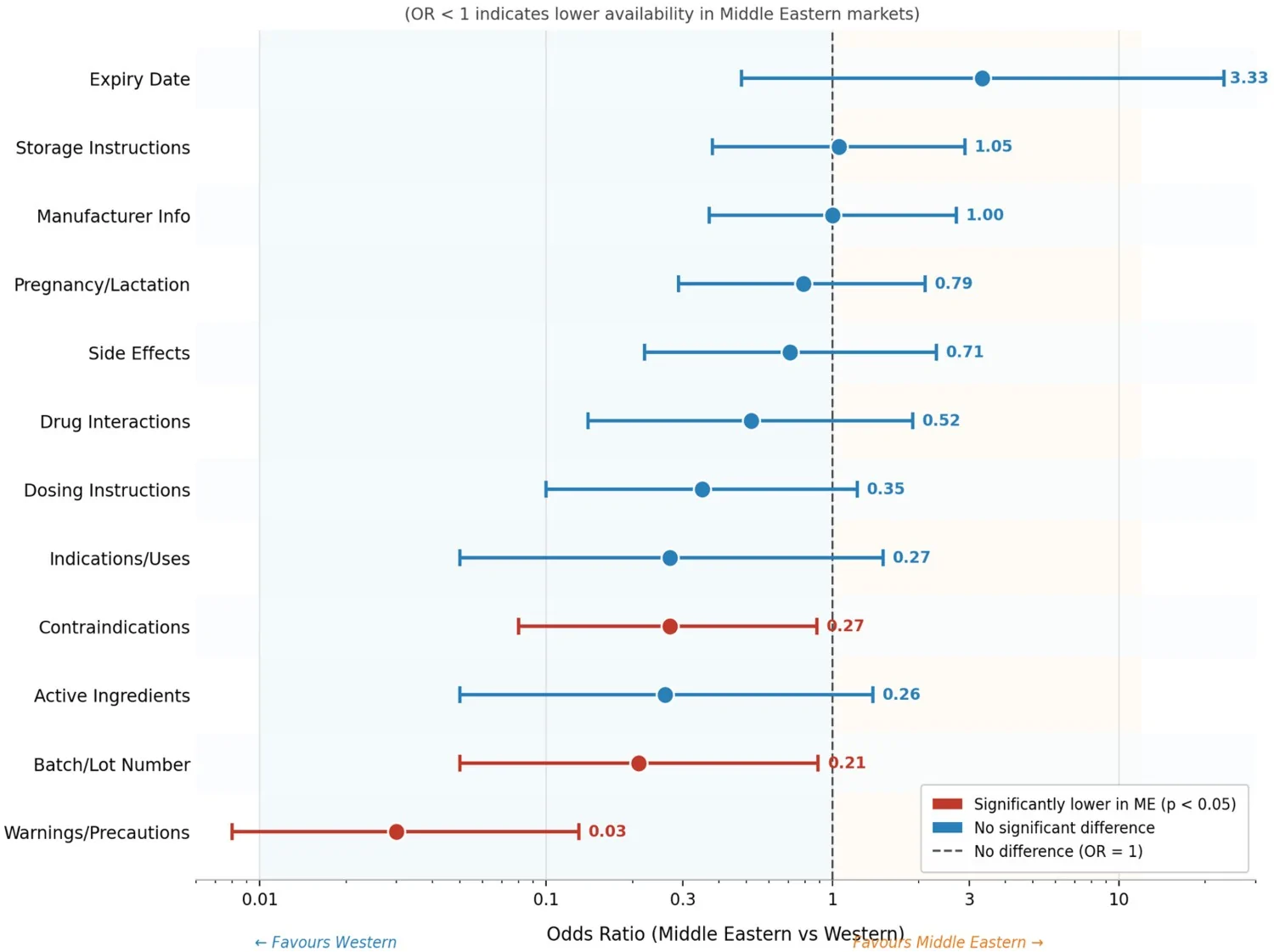
Forest plot of regional differences in safety information availability.

No other safety information element showed a statistically significant regional difference after adjustment. Effect estimates for remaining elements clustered around the null, with wide confidence intervals reflecting within-region variability (Figure 4).

### 3.5. Transparency by product category

Overall transparency did not differ across women’s health product categories. The Kruskal–Wallis H test showed no evidence of category-level variation (H(5) = 3.14, *p* = 0.679).

Median Transparency Scores across categories were similar, with substantial within-category variability. Although pregnancy and prenatal products showed slightly higher central values, these differences were not statistically meaningful.

### 3.6. Predictors of transparency score

Multivariable linear regression was used to examine predictors of the Transparency Score (Table 2). The model was statistically significant (F(7,111) = 2.11, *p* = 0.041) but explained a modest proportion of variance (adjusted R² = 0.07).

**Table 2.** Multiple linear regression predicting Transparency Score.

Predictor	B	SE	t	*p* value	95% CI
Intercept	6.32	0.43	14.73	<0.001	5.47, 7.17
Western market (vs Middle Eastern)	0.82	0.37	2.24	0.027	0.09, 1.54
Menopausal health	−0.83	0.47	−1.75	0.084	−1.77, 0.11
Pregnancy & prenatal	−0.11	0.50	−0.22	0.826	−1.10, 0.88
Reproductive health	−0.83	0.58	−1.43	0.156	−1.98, 0.32
Women’s supplements	−0.60	0.48	−1.25	0.213	−1.54, 0.35
Independent/regional platform	−0.97	0.42	−2.33	0.021	−1.79, −0.15
Online-only platform	0.14	0.53	0.27	0.792	−0.92, 1.20

Platform type emerged as the strongest predictor. Products sold through independent or regional platforms had significantly lower Transparency Scores than those sold through chain pharmacies (B = −0.97, *p* = 0.021). Market region and product category were not independently associated with transparency after adjustment.

### 3.7. Predictors of pregnancy and lactation guidance

Overall, 63.0% of products included some form of pregnancy and lactation guidance. In logistic regression analyses (Table 3), product category was the dominant predictor. Products marketed for pregnancy and prenatal use were significantly more likely to include guidance than menstrual health products (OR = 8.70, 95% CI 1.63–46.46, *p* = 0.011).

**Table 3.** Logistic regression predicting pregnancy and lactation guidance.

Predictor	OR	95% CI	*p* value
Western market	1.34	0.56–3.22	0.513
Menopausal health	0.87	0.29–2.62	0.809
Pregnancy & prenatal	8.70	1.63–46.46	0.011
Reproductive health	1.13	0.30–4.29	0.863
Women’s supplements	0.92	0.31–2.73	0.886
Online-only platform	3.15	0.61–16.32	0.172
Independent/regional platform	0.43	0.16–1.19	0.106

Platform type showed a non-significant directional association, with independent/regional platforms less likely to provide guidance (OR = 0.43, *p* = 0.106). Market region was not independently associated with the presence of pregnancy and lactation guidance.

### 3.8. Supplementary digital resources

Supplementary safety resources were inconsistently available. A downloadable patient information leaflet was present for 23.7% of products, while interactive decision-support tools were available for 40.3%. Availability did not differ significantly by region.

Products accompanied by a leaflet showed higher median Transparency Scores than those without, although this difference narrowly missed statistical significance (U = 1556.0, *p* = 0.055).

### 3.9. Expanded model including supplementary resources

An expanded regression model incorporating supplementary digital resources is summarized in Table 4. The model was statistically significant (F(9,110) = 2.13, *p* = 0.033) with R² = 0.148 and adjusted R² = 0.079.

**Table 4.** Expanded regression model predicting Transparency Score.

Predictor	B	SE	t	*p*	95% CI
Constant	5.82	0.57	10.26	< .001	[4.70, 6.94]
Western region (ref: Middle Eastern)	0.55	0.53	1.02	.308	[−0.51, 1.60]
Independent/Regional platform (ref: Chain)	−0.33	0.61	−0.54	.594	[−1.53, 0.88]
Online-only platform (ref: Chain)	0.51	0.52	0.96	.338	[−0.54, 1.55]
Menopausal health (ref: Menstrual)	−0.77	0.47	−1.63	.107	[−1.70, 0.17]
Pregnancy & prenatal (ref: Menstrual)	−0.20	0.50	−0.40	.690	[−1.18, 0.79]
Reproductive health (ref: Menstrual)	−0.57	0.59	−0.97	.333	[−1.75, 0.60]
Women’s supplements (ref: Menstrual)	−0.41	0.49	−0.85	.400	[−1.38, 0.55]
Patient information leaflet available	**0.79**	0.40	2.00	.048*	[0.01, 1.58]
Interactive decision tool available	0.68	0.35	1.97	.051	[−0.00, 1.37]

The availability of a downloadable patient information leaflet was the only predictor reaching conventional statistical significance (B = 0.79, SE = 0.40, *p* = 0.048). The interactive decision-support tool showed a borderline positive association (B = 0.68, *p* = 0.051). Market region, platform type, and product category were not independently associated with transparency in the adjusted model.

## 4. Discussion

### 4.1. Principal findings

This study provides the first cross-market quantitative assessment of safety information transparency for women’s health over-the-counter (OTC) products sold through licensed e-pharmacy platforms in Western and Middle Eastern markets. The central finding is that substantial informational incompleteness characterizes the digital retail environment for these products. With a mean Transparency Score of 6.25 out of 12, consumers were exposed, on average, to only half of the safety elements required for informed OTC self-care. Importantly, this shortfall reflects a systemic limitation in how safety-critical information is presented at the point of online purchase rather than isolated deficiencies attributable to individual retailers.

By focusing exclusively on licensed platforms, the study demonstrates that transparency gaps are embedded within regulated digital pharmacy systems and are not confined to illicit sellers, challenging the prevailing binary framing of online pharmacy risk. The findings further identify platform-level characteristics as key determinants of transparency, highlighting the influence of organizational capacity and digital infrastructure. The documented regional disparity in the availability of warnings and precautions provides empirical evidence that regulatory heterogeneity translates into unequal consumer protection. Finally, the independent association between downloadable patient information leaflets (PILs) and higher transparency identifies a concrete and actionable mechanism for improving digital medication safety.

### 4.2. Supplementary digital resources and information structure

The strong association between supplementary digital safety resources – particularly downloadable PILs – and higher Transparency Scores represents one of the most policy-relevant findings of this study. After adjustment for market region, platform type, and product category, PIL availability emerged as the strongest predictor of transparency. This reframes transparency not merely as the inclusion of discrete safety statements, but as a function of how information is organized, consolidated, and delivered within digital retail environments.

This observation aligns with evidence that consumers actively consult PILs as primary sources of medication guidance. A large-scale survey from Saudi Arabia reported that over 90% of respondents read PILs, with most indicating improved adherence and confidence in safe use (Al Jeraisy et al., 2023). The present findings extend this literature by showing that PIL availability also reflects broader informational robustness at the platform level.

The borderline association observed for interactive decision-support tools further suggests that dynamic, user-centered features may enhance transparency by contextualizing safety information and supporting decision-making. Prior work indicates that such tools can improve comprehension, particularly among older adults and individuals with limited health literacy (Martin-Hammond et al., 2015). Although not a definitive predictor in this analysis, the observed trend underscores their potential relevance as e-pharmacy interfaces continue to evolve.

### 4.3. Persistent gaps in core safety information

Despite the contribution of supplementary resources, the overall level of transparency remains concerning. The near-universal absence of expiry dates, batch or lot numbers, and drug interaction information constitutes a critical gap in consumer protection. These elements are fundamental to pharmacovigilance and risk mitigation, supporting traceability, recall identification, and anticipation of clinically significant interactions. Their systematic omission removes safeguards that are routine in physical pharmacy practice and converts online convenience into a potential public health liability.

Element-level analyses revealed particularly pronounced deficits in warnings, contraindications, and interaction information among products sold through Middle Eastern platforms. Similar shortcomings have been documented in prior evaluations of OTC and herbal products, where substantial proportions lack essential safety messaging (Raynor et al., 2011). The present study extends these concerns to women’s health OTC medicines, indicating that the continued classification of nonprescription products as inherently ‘low risk’ remains embedded in digital retail practices, despite well-established sex-specific adverse drug reaction risks (Alshammari et al., 2017; Hendriksen et al., 2021).

These deficiencies are compounded by population-level limitations in health literacy. Evidence consistently shows that many consumers struggle to interpret medication labels and safety instructions, even when information is present (Davis et al., 2006). In digital environments where safety information is incomplete or fragmented, responsibility for risk assessment shifts disproportionately to consumers, undermining equity and patient-centered care.

### 4.4. Regulatory and platform-level determinants

A key contribution of this study lies in its demonstration of cross-market disparities in safety information provision. Although differences in overall Transparency Scores were modest, the significantly lower availability of warnings and precautions in Middle Eastern platforms has direct implications for consumer safety. Warnings are the primary mechanism for alerting users to non-obvious risks and conditions requiring professional consultation; their absence represents a critical failure of risk communication.

These findings support prior characterizations of pharmacovigilance systems across Arab countries as heterogeneous and variably developed (Alshammari et al., 2019; Alshammari et al., 2020; Wilbur, 2013) and link these systemic limitations to measurable deficiencies in consumer-facing information within e-pharmacy environments. The rapid expansion of online pharmacy use in the Gulf region has outpaced the development of harmonized regulations governing digital medicine sales (Alfageh et al., 2024). In contrast, Western markets operate within more mature regulatory ecosystems with established expectations for safety disclosure (Alshammari et al., 2017).

Beyond regulatory context, platform type independently shaped transparency. Products sold through independent or regional pharmacy platforms consistently demonstrated lower Transparency Scores than those offered by large chain pharmacies. This challenges the dominant focus on illegitimate sellers as the primary source of online pharmacy risk and highlights meaningful variation within the regulated sector itself. Differences in organizational structure, technical capacity, and standardization of practice between chain and independent pharmacies likely contribute to these disparities (Nind et al., 2022), indicating that licensing alone is insufficient to ensure uniform digital pharmacy practice.

Specifying these frameworks helps locate where current rules stop short. In the United States, OTC labeling follows the Drug Facts standard at 21 CFR 201.66, while online supply is overseen through voluntary NABP Digital Pharmacy accreditation (National Association of Boards of Pharmacy, 2024; U.S. Food and Drug Administration, 2020). The United Kingdom couples the Human Medicines Regulations 2012 with the General Pharmaceutical Council’s distance-selling standards and MHRA enforcement (General Pharmaceutical Council, 2025; Medicines and Healthcare products Regulatory Agency, 2024). Australia routes every OTC product through the Therapeutic Goods Administration’s Australian Register of Therapeutic Goods and applies a standardized Consumer Medicine Information template, compliance with which became mandatory for all registered medicines from 30 December 2025 (Therapeutic Goods Administration, 2024); Canada relies on the Food and Drugs Act enforced through Health Canada and provincial registrars. In the Gulf, Saudi Arabia’s Law of Pharmaceutical Establishments and Products (Royal Decree M/108, 2023), enforced by the SFDA and supported by the Drug Track and Trace System, prescribes bilingual Arabic–English labeling and lot-level traceability (Saudi Food and Drug Authority, 2023). The UAE has restructured oversight under Federal Decree-Law No. 38 of 2024, transferring marketing-authorization functions to the Emirates Drug Establishment while community pharmacies remain with MOHAP (Ministry of Health and Prevention, 2025), and Qatar’s Ministry of Public Health licenses e-pharmacies through its Pharmacy and Drug Control Department, recognizing GCC central registration on a fast-track basis (Ministry of Public Health Qatar, 2023). Across all seven jurisdictions, none currently mandates that the safety elements assessed here appear on the consumer-facing product page; disclosure remains a platform-level decision, which our data show varies even within a single regulated market.

### 4.5. Women’s health OTC products and reproductive safety

The focus on women’s health OTC products highlights a particularly vulnerable domain within the digital marketplace. Products explicitly marketed for pregnancy and prenatal use were substantially more likely to include pregnancy and lactation guidance, demonstrating that retailers can provide reproductive safety information when it is perceived as central to product identity. However, the absence of comparable guidance across other women’s health categories suggests an implicit assumption that reproductive risk is category-specific rather than user-specific.

This assumption conflicts with real-world patterns of OTC medicine use. Women of reproductive age routinely use products across multiple categories, and prior research shows that pregnant individuals often perceive available OTC safety information as insufficient and difficult to interpret (Koo et al., 2003; Thiruchelvam et al., 2025). Regulatory gaps further exacerbate this issue, as many OTC products remain outside mandatory pregnancy and lactation labeling requirements in online contexts. The present findings quantify the consequences of this omission, demonstrating that reproductive safety information is inconsistently and voluntarily provided.

International practice already points to a workable response. The Joint FIP/WHO Good Pharmacy Practice standards expect consumer-facing information to be evidence-based, accessible, and sufficient for informed decision-making – an obligation unchanged by the move to a website (World Health Organization & International Pharmaceutical Federation, 2011). The EU Falsified Medicines Directive (2011/62/EU) and the EMA electronic Product Information program illustrate how authorized labeling can travel between physical and digital channels (European Commission, 2014; European Medicines Agency, 2023). For women’s health products specifically, extending Drug Facts-equivalent statements, advisory warnings such as those required under Australia’s RASML, and bilingual patient leaflets to the product page – and applying consistent pregnancy and lactation labeling across all categories rather than only prenatal-marketed lines – would carry existing carton-level protections into the digital point of purchase, where our findings suggest they most often disappear.

### 4.6. Implications for policy and Practice

Three concrete steps follow from these findings. The first is regulatory. Each of the seven authorities studied here already enforces detailed labeling rules at the carton or leaflet level; what is missing is a parallel obligation at the product page itself. Defining a minimum set of safety elements that platforms must display – and treating the online listing as part of the regulated unit rather than a separate retail surface – would close the most consequential gap our data identify, particularly for pregnancy and lactation guidance, which at present appears reliably only when the product is explicitly marketed as prenatal.

The second step is operational. The strong association we observed between PIL availability and transparency suggests that platforms can move ahead of regulation. Linking each product to its authorized leaflet, surfacing warnings and contraindications above the purchase action rather than inside collapsed sections, and prompting reproductive-safety review at checkout are low-cost design changes within reach of most operators. Independent and regional pharmacies, which scored lowest on most indicators, would benefit most from sector-level templates or shared content infrastructure that larger chains already deploy.

The third step concerns pharmacy practice. Pharmacists supervising distance-selling operations should treat the product page as a counseling surface and audit it against the same standards that govern in-person dispensing. Continuing professional development and undergraduate pharmacy curricula – particularly in markets where digital pharmacy is expanding most rapidly – should integrate digital health literacy and online medication-safety communication as core competencies rather than peripheral topics, equipping the next generation of pharmacists to govern the digital storefront as actively as the physical one.

### 4.7. Conclusion

Overall, this study demonstrates that safety information transparency for women’s health OTC products in e-pharmacy environments remains structurally inadequate, even within licensed platforms. Transparency deficits are patterned rather than incidental and are shaped by platform capacity, regulatory context, and the availability of supplementary resources rather than legality alone. The consistent association between downloadable PILs and higher transparency indicates that these gaps are remediable and reflect governance and design choices rather than technical constraints.

As e-pharmacies increasingly function as primary decision environments for OTC self-care, ensuring comprehensive, standardized, and accessible safety information at the point of purchase is essential. Addressing current deficiencies will require coordinated regulatory action, strengthened platform accountability, and patient-centered digital information design to support safer and more equitable online medication use. Embedding mandatory product-page disclosure into national pharmacy law – guided by the FIP/WHO Good Pharmacy Practice framework and enforced through the agencies already in place across the seven markets – offers a feasible route to closing the gap this study documents.

## Supplementary Material

Supplementary_File_1_Search_Strategy_and_Eligibility.docx
