# Supplementary material for: Safety without the counter: transparency of online safety information for women’s over-the-counter medicines across markets
Source: J Pharm Policy Pract. 2026 Aug 28;19(1):2707406. doi: 10.1080/20523211.2026.2707406 (PMC13528127; doi:10.1080/20523211.2026.2707406)
Supplement: Supplementary file 1 [file JPPP_A_2707406_SM3969.docx]

# Supplementary File 1: Search Strategy and Eligibility Criteria

This supplementary file provides a detailed, reproducible description of the search strategy and eligibility assessment used in the Women’s Health Over-the-Counter (OTC) Products Transparency Study. All procedures described here were implemented exactly as specified in the study protocol. Data collection and screening were conducted by the main investigator.

## 1. Overview of Search and Screening Process

The study employed a structured, stepwise approach to identify eligible women’s health OTC products sold through licensed e-commerce pharmacy platforms. The process consisted of two sequential phases: (1) platform eligibility verification and (2) product-level eligibility screening. Only platforms and products meeting all predefined inclusion criteria and none of the exclusion criteria were included.

## 2. Platform Eligibility Assessment

Each candidate platform was first assessed to confirm that it qualified as an e-commerce pharmacy platform. Platforms were required to demonstrate licensing or regulatory registration, active e-commerce functionality, direct-to-consumer sales, and availability in English and/or Arabic. Platforms failing any inclusion criterion or meeting any exclusion criterion were excluded prior to product screening.

| # | Inclusion Criterion | Verification Method |
| --- | --- | --- |
| 1 | Licensed or registered pharmacy | Presence of license number or regulatory logo |
| 2 | Functional e-commerce system | Add-to-cart and checkout functionality |
| 3 | English or Arabic language availability | Language selector or interface language |
| 4 | Women’s health OTC products sold | Relevant categories or searchable products |
| 5 | Dedicated product pages | Product-specific URLs with descriptions |
| 6 | Active and operational website | Website loads and allows purchases |
| 7 | Direct sales model | No redirection to third-party sellers |

## 3. Product Search Strategy

Eligible platforms were systematically searched using a standardized combination of category browsing and keyword-based searches. Category browsing was used as the primary approach when available, followed by structured keyword searches when necessary. Searches were conducted in English and/or Arabic, depending on platform language settings.

### 3.1 Women’s Health Categories

Products were identified across five predefined women’s health categories: menstrual health, menopausal health, pregnancy and prenatal health, reproductive health, and women-specific supplements.

### 3.2 Search Strings

Search strings were predefined to ensure consistency and reproducibility. For each category, multiple synonymous and condition-specific terms were used. At least three distinct search terms were attempted per category before concluding that no eligible products were available.

| Category | English Search Terms (examples) | Arabic Search Terms (examples) |
| --- | --- | --- |
| Menstrual Health | period pain; menstrual cramps; PMS relief | ألم الدورة الشهرية؛ تقلصات الدورة |
| Menopausal Health | menopause relief; hot flashes; black cohosh | سن اليأس؛ الهبات الساخنة |
| Pregnancy & Prenatal | prenatal vitamins; folic acid; pregnancy supplements | فيتامينات الحمل؛ حمض الفوليك |
| Reproductive Health | UTI relief; vaginal probiotics; emergency contraception | التهاب المسالك البولية؛ صحة المهبل |
| Women’s Supplements | women multivitamin; iron supplements women; biotin | فيتامينات للنساء؛ مكملات الحديد |

## 4. Product Eligibility Criteria

All identified products underwent individual eligibility screening. Products were required to be non-prescription, specifically marketed for women’s health, available for purchase at the time of screening, and presented on a dedicated product page in English or Arabic.

| # | Inclusion Criterion | Assessment Approach |
| --- | --- | --- |
| 1 | OTC status | Confirmed no prescription requirement |
| 2 | Women-specific indication | Product name/description specifies women |
| 3 | Oral or topical formulation | Tablets, capsules, liquids, creams |
| 4 | English or Arabic product page | Readable information in EN or AR |
| 5 | In-stock and purchasable | Add-to-cart available |
| 6 | Dedicated product page | Unique URL per product |

## 5. Eligibility Decision Workflow

Eligibility decisions followed a fixed sequence. Platforms were screened first. Products from eligible platforms were then assessed individually. Each excluded product was documented with a specific exclusion reason to ensure auditability and transparency.

Step 1: Verify platform eligibility against all inclusion and exclusion criteria.
Step 2: Identify candidate products using category browsing and predefined search strings.
Step 3: Apply product-level inclusion and exclusion criteria.
Step 4: Include eligible products and extract transparency data; exclude ineligible products with justification.

## 6. Reproducibility and Quality Control

To ensure reproducibility, the same search strings, language settings, and eligibility rules were applied consistently across all platforms. Screening decisions were made by the main investigator using a standardized screening log. Ambiguous cases were resolved by referring back to the predefined criteria rather than subjective judgment.
